# IVUS‐Guided Versus Angiography‐Guided PCI for Unprotected Left Main Coronary Artery Disease: A Systematic Review, Meta‐Analysis, and GRADE Assessment of Randomized Trials

**DOI:** 10.1002/clc.70367

**Published:** 2026-06-10

**Authors:** Abdelrahman Elhakim, Mostafa Salem, Mohammed A. Elbahloul, Abdelrahman M. Elettreby, Mohamed A. Alsaied, Hamza A. Abdul‐Hafez, Ghaith Hussein Ahmad Al Bataineh, Maab M. Saleh, Amer Al Manla, Osama Bisht

**Affiliations:** ^1^ Department of cardiology and intensive care Städtisches Klinikum Braunschweig Braunschweig Germany; ^2^ Department of Internal Medicine III Cardiology, Angiology and Intensive Care Medicine, University Hospital Schleswig‐Holstein Kiel Germany; ^3^ Faculty of Medicine Kafr El‐Shaikh University Kafr El Shaikh Egypt; ^4^ Faculty of Medicine Mansoura University Mansoura Egypt; ^5^ Faculty of Medicine An‐Najah National University Nablus Palestine; ^6^ Internal Medicine Resident—Jordan University of Science and Technology Irbid Jordan; ^7^ Evanglisches Herzzentrum Coswig Coswig Germany

**Keywords:** coronary artery diseases, intravascular ultrasound, left main coronary artery disease, percutaneous coronary intervention, unprotected left main coronary artery

## Abstract

**Background:**

Intravascular ultrasound (IVUS) has been increasingly used as an adjunctive tool for complex percutaneous interventions (PCIs); however, comparative randomized evidence with conventional angiography in unprotected left main coronary artery (ULMCA) disease remains scarce and fragmented. Therefore, this systematic review and meta‐analysis aimed to assess and synthesize evidence regarding its use in ULMCA disease.

**Methods:**

We performed a systematic literature search across PubMed, Scopus, Web of Science, and Cochrane until April 2026 to identify relevant RCTs comparing IVUS‐guided PCI with conventional angiography‐guided PCI in ULMCA disease. Risk of bias of studies was assessed using the Cochrane RoB‐2 tool. A random‐effects model meta‐analysis was performed in R, with an exploratory univariate meta‐regression of covariates.

**Results:**

Four randomized trials involving 2278 patients with ULMCA disease were included. Compared with angiography‐guided PCI, IVUS guidance was associated with numerically lower risks of all‐cause death, cardiac death, target lesion revascularization, and target vessel revascularization, while myocardial infarction and stent thrombosis were similar between groups.

**Conclusion:**

In patients undergoing PCI for ULMCA disease, IVUS guidance was associated with numerically favorable but statistically nonsignificant reductions in mortality and repeat revascularization, without clear differences in myocardial infarction or stent thrombosis. Although randomized evidence is still lacking to determine clinical superiority, these results justify powered future trials to determine whether the effects of IVUS guidance vary according to prior myocardial infarction status and left ventricular ejection fraction.

## Graphical abstract

graphical abstract for IVUS‐guided versus angiography‐guided pci for unprotected left main coronary artery disease.

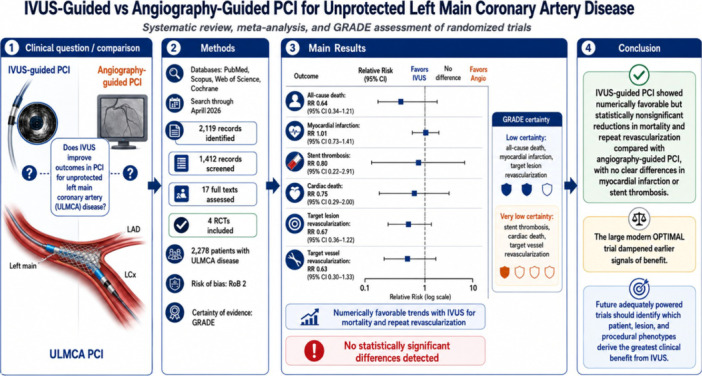

**Abbreviations**
IVUS: intravascular ultrasound
PCI: percutaneous catheter intervention

## 1 Introduction

Unprotected left main coronary artery (ULMCA) disease is one of the most prognostically significant manifestations of obstructive coronary artery disease, as it threatens a significant myocardial area and often requires revascularization [1]. Despite the fact that the use of coronary artery bypass grafting has traditionally been viewed as the reference strategy, modern guidelines have come to view percutaneous coronary intervention (PCI) as a reasonable form of revascularization in selected patients, especially in cases where anatomic complexity is not restrictive and the use of multidisciplinary decision‐making in favor of a percutaneous approach [2, 3]. This development has increased interest in adjunctive procedural strategies that can enhance the safety, accuracy, and longevity of left main PCI [4].

Intravascular ultrasound (IVUS) is one such strategy, which is especially appealing because it overcomes the inherent drawbacks of two‐dimensional angiography by directly assessing plaque distribution, vessel size, lesion length, calcium load, landing zones, and post‐stent expansion or malapposition [5, 6]. These features are particularly pertinent in ULMCA PCI, in which large‐vessel caliber, ostial disease, distal bifurcation anatomy, and the clinical implications of underexpansion can significantly affect outcomes [7]. Consistent with this mechanistic explanation, there is now guideline and consensus support for intracoronary imaging: the 2024 European Society of Cardiology guideline of chronic coronary syndromes has elevated intravascular imaging to a class I recommendation in anatomically complex PCI, including left main lesions, and the 2021 ACC/AHA/SCAI revascularization guideline has found that use of imaging‐guided PCI has increased in contemporary practice, although uptake remains heterogeneous across regions and health systems [4, 8]. Meanwhile, imaging‐guided PCI has become more common in modern practice, although its adoption is uneven across regions and health systems [9, 10].

Regardless of this trend, the disease‐specific evidence base of routine IVUS during ULMCA PCI is yet to be established. Meta‐analyses of large populations of PCI patients have generally indicated that intravascular imaging guidance is linked with a reduced incidence of cardiac death, stent thrombosis, target lesion revascularization, and other ischemic outcomes [11, 12], and that updated network analyses have indicated that the benefit may be strongest with respect to repeat revascularization and may be more consistent with IVUS than angiography [13, 14]. Nevertheless, such pooled datasets include heterogeneous lesion subsets, imaging modalities, and patient‐risk profiles and thus cannot definitively answer whether the benefits of complex PCI directly apply to the unique anatomy and procedural requirements of unprotected left main intervention.

Randomized evidence specific to ULMCA PCI has also evolved unevenly. Previous left‐main‐centered trials implied reduced incidences of composite ischemic events or repeat revascularization with IVUS guidance [15], and the prespecified left‐main analysis of RENOVATE‐COMPLEX‐PCI extrapolated this message to intravascular imaging‐guided PCI [16]. Even more recently, a modern randomized trial in Egypt once again found IVUS‐guided PCI to be superior [17], but the largest randomized trial to date in ULMCA disease, the OPTIMAL trial, did not show a superiority of standard IVUS guidance to the patient‐oriented composite endpoint [18]. These ostensibly discordant data, combined with a growing focus on imaging in the guidelines on complex PCI, create a clear need for a focused synthesis limited to randomized evidence in ULMCA disease. Based on this, we conducted a systematic review and meta‐analysis of randomized controlled trials (RCTs) comparing IVUS‐guided PCI with angiography‐guided PCI in patients with ULMCA disease. We aimed to measure the impact of IVUS guidance on mortality, ischemic events, stent thrombosis, repeat revascularization, and procedure duration, and to evaluate the confidence in the evidence using the GRADE framework.

## 2 Methods

### 2.1 Protocol and Registration

Before conducting this study, a protocol was prospectively registered with PROSPERO (CRD420261387817). This study was conducted in accordance with the Preferred Reporting Items for Systematic Reviews and Meta‐Analyses (PRISMA) statement and Cochrane Handbook for Systematic Reviews of Interventions [19, 20].

### 2.2 Eligibility Criteria

#### 2.2.1 Inclusion Criteria

We incorporated in the systematic review and quantitative synthesis studies fulfilling the following PICOs criteria; (i) population: adult patients (≥18 years) undergoing PCI for angiographically confirmed ULMCA disease/lesions (defined as visually estimated ≥ 50% left main stenosis); (ii) intervention: IVUS‐guided PCI; (iii) comparator: conventional angiography‐guided PCI without planned IVUS assessment; (iv) outcomes: the prespecified outcomes of this review were all‐cause death, myocardial infarction, and stent thrombosis as primary endpoints, with cardiac death, target lesion revascularization, and target vessel revascularization evaluated as secondary endpoints, and (v) study designs: only comparative RCTs were included.

#### 2.2.2 Exclusion Criteria

We excluded non‐randomized studies, including observational studies, case reports, case series, reviews, commentary articles, and abstracts with non‐extractable results. We also excluded studies that enrolled patients with coronary artery disease other than ULMCA, non‐comparative studies, studies comparing IVUS to other interventions other than angiographic PCI, and studies with no extractable outcome results.

### 2.3 Information Sources and Search Strategy

We performed a literature search across PubMed, Scopus, Cochrane and Web of Science from inception till April 2026 utilizing the following search strategy: (IVUS OR intravascular ultrasound OR intravascular imaging OR intravascular ultrasonography OR IVUS‐Guided) AND (PCI OR Percutaneous Coronary Intervention OR stent) AND (left main coronary artery OR LMCA)], adapted to each database and limited to English studies only with no additional filters. Supporting Information S1: Table 1 in the Supporting Information Materials details the search strategies used for each database search.

### 2.4 Study Selection and Data Extraction

#### 2.4.1 Study Selection Process

All records from the database search were retrieved and imported into EndNote [21] to remove duplicate records. After duplicates were removed, the records were screened against the predefined eligibility criteria using the Rayyan platform [22]. Titles and abstracts were screened to remove irrelevant records, and then the full texts of potentially eligible records were retrieved. Full texts were examined against the eligibility criteria to identify the final included studies. Screening was performed by two independent authors, with a third author's opinion in the event of discrepancies in judgment.

#### 2.4.2 Data Extraction Process

Two authors independently utilized a standardized data collection form in extracting data from each included trial. The variables that were extracted were study identifiers and design characteristics, country, sample size, follow‐up duration, type of stent, and trial‐specified primary outcome. Moreover, when available, baseline patient characteristics were obtained, such as age, sex, hypertension, dyslipidemia, diabetes mellitus, current smoking, previous myocardial infarction, previous PCI, and the left ventricular ejection fraction. Outcome data pertinent to the predetermined endpoints of the review were also tabulated based on the original definitions as reported in each trial.

### 2.5 Risk of Bias Assessment

Two reviewers independently assessed the risk of bias of the included trials using the Cochrane Risk of Bias 2 (RoB 2) tool across the domains of randomization, deviations from intended interventions, missing outcome data, outcome measurement, and selection of the reported result. Each study was classified as low risk of bias, some concerns, or high risk of bias [23].

The certainty of evidence for the prespecified outcomes was evaluated using the GRADE framework and categorized as high, moderate, low, or very low after consideration of risk of bias, inconsistency, indirectness, imprecision, and publication bias [24].

### 2.6 Statistical Analysis and Heterogeneity

Data were pooled using a random‐effects meta‐analysis to account for anticipated between‐study variability in patient characteristics. For dichotomous outcomes, effect estimates were expressed as risk ratios (RR) with 95% confidence intervals (CI), whereas continuous outcomes were summarized using mean differences (MD) with 95% CI. Statistical heterogeneity was assessed using Cochran's *Q* test and quantified with the *I* ^2^ statistic, with higher *I* ^2^ values indicating greater inconsistency across studies. A leave‐one‐out sensitivity analysis was performed for each pooled outcome to evaluate the robustness of the summary estimates and to determine whether any single study disproportionately influenced the overall effect. Publication bias was explored using Doi plots and the Luis Furuya‐Kanamori (LFK) index [25], with greater deviation from zero indicating increasing asymmetry. In addition, trim and fill correction was applied. A two‐sided *p* value < 0.05 was considered statistically significant. The analysis was conducted using R software [26].

## 3 Results

### 3.1 Study Selection

**Figure 1:**
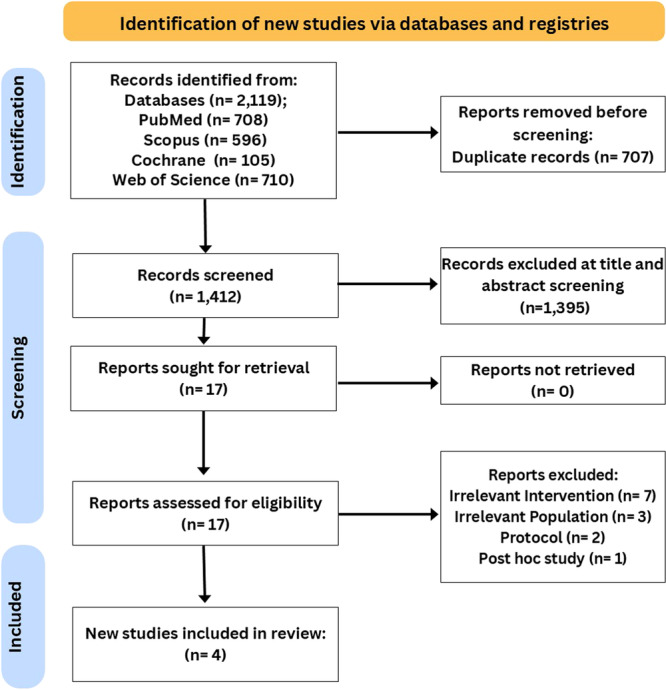
PRISMA flow diagram illustrating the study selection process.

A total of 2119 records were identified through database searching, including PubMed (*n* = 708), Scopus (*n* = 596), Cochrane (*n* = 105), and Web of Science (*n* = 710). After removal of 707 duplicates, 1412 records underwent title and abstract screening, of which 1395 were excluded. Seventeen reports were sought for retrieval, and all were assessed for eligibility. Of these, 13 reports were excluded, leaving four randomized trials [15, 17, 18, 27] included in the systematic review. Study selection is illustrated in Figure 1.

### 3.2 Characteristics of Included Studies

A total of four randomized trials were included, enrolling 2278 patients with ULMCA disease/lesions undergoing PCI across Egypt, China, Korea, and Europe; the study samples ranged from 123 to 806 patients, and follow‐up ranged from 1 to 3 years. The majority of trials were single‐ or multicenter RCTs. The populations included those with angiographically or visually verified ≥50% left main stenosis, and one trial was limited to the elderly population (>70 years old) [15]. All were done with drug‐eluting stents, such as sirolimus‐eluting and everolimus‐eluting platinum‐chromium. Across studies, mean age was generally in the mid‐60s to early 70 s; most participants were men, and hypertension, diabetes, prior MI, and prior PCI were common baseline comorbidities. Table 1 shows a summary of the included studies, and Table 2 details the baseline characteristics of the included studies.

**Table 1:** Summary of included studies.

Study	Study design, region	Country	Sample size (IVUS vs Angio)	Follow‐up duration	Stent type	Primary outcome	Patients
Bendary et al. [17]	Two‐center, RCT	Egypt	181 (90 vs 91)	1 year	DES	MACE: defined as MI, TVR, and cardiac death.	Patients (18 and 75 years) with ULMCA lesions identified by visual examination as having at least 50% LMCA stenosis.
Liu et al. [27]	Single‐center, open‐label RCT	China	336 (167 vs 169)	1 year	DES	MACEs: defined as cardiac death, MI, and TVR	patients with ULMCA lesions (defined as at least 50% stenosis in the left main coronary artery from visual assessment)
Tan et al. [15]	Single‐center, RCT	China	123 (61 vs 62)	2 Years	Sirolimus‐eluting DES	MACE: defined as death, non‐fatal MI, and TLR.	Elderly patients (age > 70) with ULMCA ( defined as at least 50% stenosis by visual assessment in the LM vessel without bypass grafts to the left anterior descending artery or left circumflex artery.
Testa et al. [18]	International, multicenter, open‐label, superiority RCT	Italy, Spain, and the United Kingdom.	806 (401 vs 405)	2.9 years	Everolimus‐ eluting platinum–chromium stents	Patient‐oriented composite of any stroke, any MI, any revascularization, or death from any cause	Patients 18 years of age or older with ULMCA disease with a stenosis diameter of at least 50% (as visually estimated on angiography)

Abbreviations: DES, drug‐eluting stents; IVUS, intravascular ultrasound; MI, myocardial infarction; RCT, MACE, major adverse cardiac events; TLR, target lesion revascularization; TVR, target vessel revascularization; ULMCA, unprotected left main coronary artery.

**Table 2:** Baseline characteristics of included studies.

Study	Arms	No. of participants	Age (years), mean (SD)	Male, n (%)	Hypertension, *n* (%)	Dyslipidemia, *n* (%)	Diabetes, *n* (%)	Current smoker, *n* (%)	Prior MI, *n* (%)	Prior PCI, *n* (%)	LVEF %, mean (SD)
Bendary et al. [17]	IVUS	90	63 (9)	66 (73.3)	68 (75.6)	67 (74.4)	56 (62.2)	52 (57.8)	16 (17.8)	33 (36.7)	55 (9)
	Angiography	91	61 (9)	63 (69.2)	61 (67)	58 (63.7)	63 (69.2)	50 (54.9)	13 (14.3)	26 (28.6)	54 (8)
Liu et al. [27]	IVUS	167	65.3 (10.6)	106 (63.5)	116 (69.5)	63 (37.7)	56 (33.5)	62 (37.1)	29 (17.4)	33 (19.8)	55.6 (11.7)
	ICA	169	64.9 (11.2)	108 (63.9)	122 (72.2)	64 (37.9)	52 (30.8)	60 (35.5)	24 (14.2)	28 (16.6)	58.4 (10.5)
Tan et al. [15]	Angiography	62	75.9 (3.5)	43 (69.4)	29 (46.8)	N.A	18 (29.5)	29 (46.8)	13 (21)	NA	53.3 (7.1)
	IVUS	61	76.5 (5)	38 (62.3)	25 (41)	N.A	21 (34.4)	27 (44.3)	10 (16.4)	NA	55.3 (5)
Testa et al. [18], OPTIMAL	IVUS	401	71.1 (11.0)	315 (78.6)	318 (79.5)	296 (74.0)	147 (36.6)	52 (13.0)	100 (25.0)	105 (26.2)	53.2 (11.1)
	Angiography	405	71.7 (10.4)	317 (78.3)	321 (79.3)	315 (77.8)	133 (32.8)	61 (15.1)	90 (22.2)	100 (24.7)	53.4 (10.4)

Abbreviations: IVUS, intravascular ultrasonography; LVEF, left ventricular ejection fraction; MI, myocardial infarction; N., number; N.A, not available; PCI, percutaneous coronary intervention.

### 3.3 Risk of Bias and Quality Assessment

**Figure 2:**
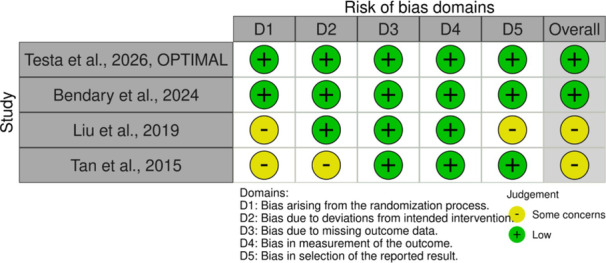
Risk of bias graph of included clinical trials using Cochrane RoB 2 tool.

Risk of bias of included RCTs shows that two RCTs (Testa et al. [18] and Bendary et al. [17]) were judged as low risk of bias, while two RCTs (Liu et al. [27] and Tan et al. [15]) showed some concerns (Figure 2).

### 3.4 Primary Outcomes

**Figure 3:**
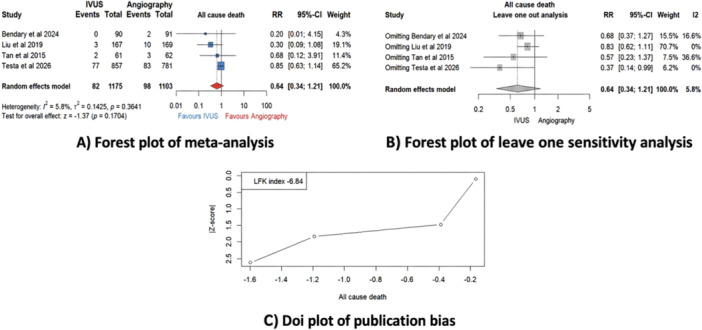
Meta‐analysis of all‐cause death comparing intravascular ultrasound‐guided (IVUS) versus angiography‐guided left main coronary artery intervention. (A) Forest plot of all‐cause death, (B) Leave‐one‐out sensitivity analysis of all‐cause death, and (C) Doi plot assessing publication bias for all‐cause death.

**Figure 4:**
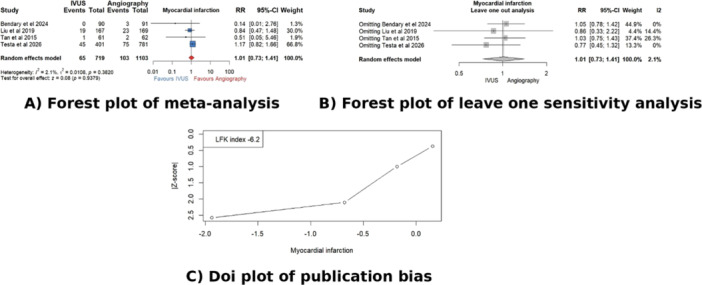
Meta‐analysis of Myocardial infarction (MI) comparing intravascular ultrasound‐guided (IVUS) versus angiography‐guided left main coronary artery intervention. (A) Forest plot of Myocardial infarction, (B) Leave‐one‐out sensitivity analysis of Myocardial infarction, and (C) Doi plot assessing publication bias for Myocardial infarction.

**Figure 5:**
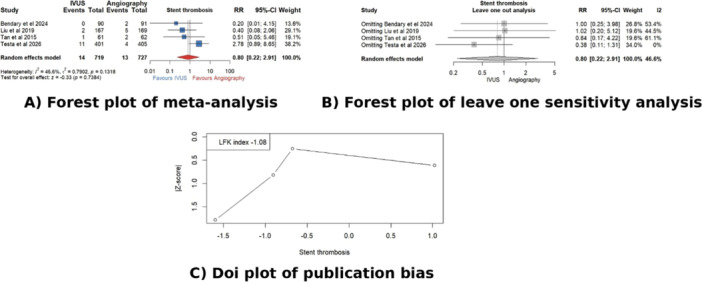
Meta‐analysis of stent thrombosis comparing intravascular ultrasound‐guided (IVUS) versus angiography‐guided left main coronary artery intervention. (A) Forest plot of stent thrombosis, (B) Leave‐one‐out sensitivity analysis of stent thrombosis, and (C) Doi plot assessing publication bias for stent thrombosis.

For the primary outcomes, pooled analysis showed that IVUS‐guided left main coronary artery intervention was associated with a numerically lower risk of all‐cause death compared with conventional angiography, although this did not reach statistical significance (RR = 0.64, 95% CI: 0.34–1.21; *p* = 0.17), with no heterogeneity across studies (*I* ^2^ = 5.8%) (Figure 3A). The leave‐one‐out sensitivity analysis demonstrated that the direction and magnitude of the effect remained generally consistent after sequential omission of individual studies, suggesting acceptable robustness overall, although omission of Testa et al. [18] appeared to strengthen the association (Figure 3B). Myocardial infarction was comparable between the two groups (RR = 1.01, 95% CI: 0.73–1.41; *p* = 0.94), with no observed heterogeneity (*I* ^2^ = 2.1%) (Figure 4A), and the leave‐one‐out analysis confirmed a highly stable result **(**Figure 4B **)**. Likewise, no significant difference was observed for stent thrombosis (RR = 0.8, 95% CI: 0.22–2.91; *p* = 0.74), with moderate heterogeneity (*I* ^2^ = 46.6%) (Figure 5A); sensitivity analysis showed no single study significantly altered the overall result (Figure 5B). Publication bias assessment using Doi plots suggested major asymmetry for all‐cause death (LFK index = −6.84) (Figure 3C) and myocardial infarction (LFK index = −6.2) (Figure 4C), and mild asymmetry for stent thrombosis (LFK index = −1.08) (Figure 5C).

Trim‐and‐fill analysis further suggested possible small‐study effects, as two potentially missing studies were imputed for each assessed outcome. After adjustment, the pooled estimates remained statistically non‐significant for all‐cause death (RR 0.83, 95% CI 0.51–1.36; *p* = 0.458) (Supporting Information S1: Figure 1), myocardial infarction (RR 1.07, 95% CI 0.80–1.43; *p* = 0.666) (Supporting Information S1: Figure 2), and stent thrombosis (RR 1.77, 95% CI 0.46–6.80; *p* = 0.403) (Supporting Information S1: Figure 2). These findings indicate that although asymmetry was detected and imputed studies were added, adjustment using trim‐and‐fill did not significantly change the overall conclusions, as none of the outcomes showed a statistically significant difference between IVUS and angiography‐guided PCI.

### 3.5 Secondary Outcomes

For the secondary outcomes, IVUS‐guided intervention was associated with a numerically lower risk of cardiac death compared with angiography alone, although the difference was not statistically significant (RR = 0.75, 95% CI: 0.29–2; *p* = 0.57), with substantial heterogeneity (I^2^ = 58.6%) (Supporting Information S1: Figure 4). Leave‐one‐out analysis showed that the observed effect was sensitive to Liu et al. [18] (Supporting Information S1: Figure 5). TLR was lower with IVUS guidance but did not reach statistical significance (RR = 0.67, 95% CI: 0.36–1.22; *p* = 0.187), with mild heterogeneity (*I* ^2^ = 18.4%) (Supporting Information S1: Figure 6); leave‐one‐out analysis showed significant results when Testa et al. [18] was removed (Supporting Information S1: Figure 7). Similarly, TVR tended to be lower in the IVUS arm without achieving statistical significance (RR = 0.63, 95% CI: 0.3–1.33; *p* = 0.224), with substantial heterogeneity (*I* ^2^ = 62.9%) (Supporting Information S1: Figure 8), and sensitivity analysis demonstrated pooled estimates favoring IVUS when Testa et al. [18] was removed (Supporting Information S1: Figure 9).

Publication bias assessment using Doi plots demonstrated major asymmetry for cardiac death (LFK index = −4.26) (Supporting Information S1: Figure 10), TLR (LFK index = −5.72) (Supporting Information S1: Figure 11), and TVR (LFK index = −4.8) (Supporting Information S1: Figure 12). Trim‐and‐fill analysis further suggested possible small‐study effects, as two potentially missing studies were imputed for each assessed outcome. After adjustment, the pooled estimates remained statistically non‐significant for cardiac death (RR 1.32, 95% CI 0.50–3.54; *p* = 0.577) (Supporting Information S1: Figure 13), target lesion revascularization (RR 0.90, 95% CI 0.52–1.55; *p* = 0.698) (Supporting Information S1: Figure S14), and target vessel revascularization (RR 1.06, 95% CI 0.46–2.45; *p* = 0.886) (Supporting Information S1: Figure 15). These findings indicate that although asymmetry was detected and imputed studies were added, adjustment using trim‐and‐fill did not materially change the overall conclusions, as none of the outcomes showed a statistically significant difference between IVUS and angiography‐guided PCI. However, the interpretation should remain cautious because several outcomes showed moderate‐to‐substantial residual heterogeneity, particularly cardiac death and target vessel revascularization.

### 3.6 Certainty of Evidence

The certainty of evidence was rated as low in all‐cause death, myocardial infarction, and target lesion revascularization, and very low in stent thrombosis, cardiac death, and target vessel revascularization. The leading factor behind downgrading was imprecision, with the majority of the pooled CIs crossing the line of no effect, and they were especially broad for stent thrombosis and cardiac death. Further downgrades were made due to inconsistency in cardiac death and target vessel revascularization, and due to possible publication bias/small‐study effects in outcomes with significant Doi plot asymmetry. Conversely, risk of bias and indirectness were not deemed serious enough to justify downgrading the pooled evidence in general. Table 3 shows the detailed certainty of evidence using the GRADE framework.

**Table 3:** GRADE assessment of certainty of evidence for pooled clinical outcomes.

Outcome	Studies	Pooled effect estimate	Risk of bias	Inconsistency	Indirectness	Imprecision	Publication bias	Overall certainty	Brief explanation of judgment
**All‐cause death**	4 RCTs	RR 0.64 (95% CI 0.34–1.21); *I* ^2^ = 5.8%	Not serious	Not serious	Not serious	Serious	Serious	Low ⊕⊕◯◯	Downgraded for imprecision because the CI crossed unity; downgraded for publication bias because the Doi plot showed major asymmetry.
**Myocardial infarction**	4 RCTs	RR 1.01 (95% CI 0.73–1.41); *I* ^2^ = 2.1%	Not serious	Not serious	Not serious	Serious	Serious	Low ⊕⊕◯◯	Downgraded for imprecision because the CI crossed unity; downgraded for publication bias because the Doi plot showed major asymmetry.
**Stent thrombosis**	4 RCTs	RR 0.80 (95% CI 0.22–2.91); *I* ^2^ = 46.6%	Not serious	Not serious	Not serious	Very serious	Serious	Very low ⊕◯◯◯	Downgraded for very serious imprecision because the CI was very wide for a rare‐event outcome; downgraded for publication bias because the Doi plot showed moderate asymmetry.
**Cardiac death**	3 RCTs	RR 0.75 (95% CI 0.29–2.00); *I* ^2^ = 58.6%	Not serious	Serious	Not serious	Very serious	Serious	Very low ⊕◯◯◯	Downgraded for inconsistency because heterogeneity was substantial and sensitivity analysis showed instability; downgraded for very serious imprecision because the CI was very wide; downgraded for publication bias because the Doi plot showed major asymmetry.
**Target lesion revascularization (TLR)**	3 RCTs	RR 0.67 (95% CI 0.36–1.22); *I* ^2^ = 18.4%	Not serious	Not serious	Not serious	Serious	Serious	Low ⊕⊕◯◯	Downgraded for imprecision because the CI crossed unity; downgraded for publication bias because the Doi plot showed major asymmetry.
**Target vessel revascularization (TVR)**	3 RCTs	RR 0.63 (95% CI 0.30–1.33); *I* ^2^ = 62.9%	Not serious	Serious	Not serious	Serious	Serious	Very low ⊕◯◯◯	Downgraded for inconsistency because heterogeneity was moderate‐to‐substantial; downgraded for imprecision because the CI crossed unity; downgraded for publication bias because the Doi plot showed major asymmetry.

*Note:* All outcomes started at high certainty because only randomized controlled trials were included. Certainty was downgraded according to GRADE domains when concerns were identified regarding inconsistency, indirectness, imprecision, publication bias, or risk of bias.

## 4 Discussion

IVUS‐guided PCI for ULMCA disease in this meta‐analysis of randomized trials was associated with uniformly positive directional effects on mortality and repeat revascularization, with no apparent differences in myocardial infarction or stent thrombosis. These results are, however, inconclusive due to limitations in the pooled estimates, including imprecision, heterogeneity of the outcomes, and the significant impact of a single large current trial. This uncertainty was present in the GRADE assessment, where most outcomes were characterized by low certainty, and some clinically important endpoints had very low certainty. Collectively, the evidence at hand indicates a potential advantage of IVUS in left main PCI, but does not provide conclusive evidence of routine clinical superiority across all patient and lesion groups.

That subtlety is significant as the mechanistic argument of IVUS in left main intervention is solid. The left main artery is large, frequently bifurcates into complex distal anatomy, and is unforgiving of geographic miss, underexpansion, malapposition, or unrecognized edge dissection [28]. IVUS directly overcomes these weaknesses through better vessel sizing, selection of the landing zone, evaluation of plaque distribution, and optimization of post‐stent [29, 30]. Such considerations can be used to understand why modern expert reviews and practice statements are starting to consider intracoronary imaging as part of complex PCI, and why recent European recommendations have elevated intravascular imaging in anatomically complex PCI, such as left main disease [4]. Recent guidelines and real‐world data have also identified IVUS as a tool that can be used to assess left main lesions and optimize procedures [8, 31].

We must interpret our findings as well within the broader randomized imaging literature. In non‐left‐main and mixed‐complexity PCI populations, randomized trials and trial‐level meta‐analyses have generally favored intravascular imaging over angiography alone, especially for repeat revascularization and, in some studies, for myocardial infarction or cardiac death [11, 32]. The extension of follow‐up for ULTIMATE and IVUS‐XPL, the extensive ACS‐based IVUS‐ACS program, and the recent meta‐analyses of randomized trials have strengthened the perception that imaging‐guided PCI may frequently yield better clinically relevant outcomes than angiographic guidance [33, 34, 35]. Network meta‐analysis has also indicated that IVUS is the modality with the most established evidence base of all intracoronary imaging modalities, particularly in stent‐related outcomes [13, 14]. Consistently, a recent systematic review and meta‐analysis focused specifically on bifurcation and unprotected left main lesions found that intravascular imaging guidance reduced target vessel failure compared with angiographic guidance, reinforcing the concept that lesion complexity is a key determinant of imaging benefit [12]. On that basis, a left‐main‐specific randomized synthesis would have been anticipated to demonstrate a more apparent additive effect of IVUS than we eventually found.

Part of this is because the previous left‐main‐specific randomized studies, when combined, were in support of IVUS. Liu and Tan proposed reduced composite ischemic events, and cardiac death and revascularization became the primary domains where IVUS seemed to be most promising [15, 27]. Bendary also preferred IVUS‐guided PCI reporting procedural differences that were in line with more aggressive optimization in the imaging arm [17]. This signal was further extended by the Kwon substudy in a more modern context, demonstrating the advantage of intravascular imaging‐guided PCI in a prespecified left‐main cohort within RENOVATE‐COMPLEX‐PCI [16]. Though IVUS or OCT was permitted by Kwon, IVUS was the most common, and the optimization‐oriented design of the trial was conceptually consistent with the current question. Collectively, these studies supported the pre‐OPTIMAL assumption that left main PCI is among the lesion subsets most likely to respond to imaging guidance.

The strongest counterweight to that expectation is the OPTIMAL trial [18]. Being the largest, most rigorously randomized trial in ULMCA disease, international, and conducted in established centers with adjudicated clinical events, OPTIMAL is not just another neutral study, but a potentially practice‐reframing trial. It directly contradicts the belief that routine IVUS guidance is always necessary to provide a measurable clinical benefit to modern left main PCI. That is significant in our dataset. A leave‐one‐out analysis revealed that the exclusion of OPTIMAL (Testa et al.) moved the mortality signal further towards IVUS, and the directional signals of stent thrombosis and repeat revascularization were positive or stronger. In the case of cardiac death, especially, the pooled estimate was sensitive to OPTIMAL. That is precisely the trend that should be observed when a large, methodologically sound, neutral trial is introduced into a literature previously influenced by smaller, positive studies. OPTIMAL thus transforms the discussion to be not about whether IVUS helps or not, but about who, in what circumstances, and with what results, does IVUS make the most difference.

This discordance can be explained by several reasons. First, OPTIMAL compared standard IVUS with very modern angiography‐guided PCI conducted in highly skilled centers, which probably reduced the performance difference between the strategies. Second, it had a patient‐oriented, not device‐ or lesion‐oriented, primary endpoint; this is a clinically meaningful endpoint, but it can obscure benefits most closely associated with stent optimization. Third, OPTIMAL recruited a complicated left‐main population, whose anatomic burden was not insignificant by the standards of left‐main trials. The results in these patients can be influenced by imaging utilization, lesion morphology, bifurcation approach, full revascularization, calcium alteration, and the patient's overall risk. Fourth, technically unsophisticated PCI is no longer synonymous with modern angiography‐guided PCI [36]. These are not meant to downplay the usefulness of IVUS, but they imply that the marginal benefit of this technique might be context‐dependent and harder to demonstrate in cases where the control arm is highly optimized [37].

### 4.1 Strengths and Limitations

The study has several strengths. It was limited to randomized evidence, included the recently obtained OPTIMAL data, and was based on a framework aligned with current evidence synthesis guidelines, including systematic risk‐of‐bias evaluation, leave‐one‐out sensitivity analysis, exploration of publication bias, and GRADE certainty appraisal. These options are important because they minimize the upward bias that can arise when observational left‐main IVUS studies predominate the evidence base.

This study has several limitations. First, the number of included studies per outcome was small, limiting statistical power and reducing the reliability of publication bias assessment findings. Second, some pooled analyses showed moderate heterogeneity, particularly for cardiac death and target vessel revascularization, suggesting important between‐study differences in patient selection, procedural techniques, operator experience, stent platforms, and follow‐up duration. Third, some outcomes demonstrated marked asymmetry on the Doi plot assessment, raising the possibility of publication bias or small‐study effects. Finally, because the analysis combined a limited number of studies with varying designs and baseline risk profiles, the overall findings should be interpreted with caution, particularly for outcomes that did not reach statistical significance.

### 4.2 Implication for Clinical Practice and Future Research

Clinically, selective optimism is the most defensible conclusion as opposed to routine triumphalism. IVUS is an attractive supplement to left main PCI, and nothing in the current analysis undermines its clinical utility in terms of lesion characterization, vessel sizing, evaluation of plaque distribution, stent optimization, and identification of suboptimal expansion or edge‐related complications. This stance is also widely consistent with current recommendations: the 2024 ESC guideline supports the use of intracoronary imaging in complex PCI, such as left main disease, and the 2021 ACC/AHA/SCAI guideline acknowledges IVUS to be helpful in the assessment and optimization of left main PCI [4, 8]. However, the existing randomized evidence, especially following the introduction of OPTIMAL, does not lend credence to an unqualified belief of universal clinical superiority under normal circumstances of routine IVUS usage in all patients undergoing ULMCA intervention.

In this respect, the question of whether IVUS is conceptually useful or not is no longer the most pertinent clinical question, but how it is to be used, which kind of optimization is of the greatest importance, and who is most likely to gain a measurable benefit [38, 39]. Future research in that direction should not focus on routine‐versus‐no‐routine comparisons but rather should prospectively compare patients with distal bifurcation disease, heavy plaque burden, impaired ventricular function, prior myocardial infarction burden, or other indicators of anatomical and biological susceptibility. IVUS in ULMCA PCI must be considered as a valuable, frequently reasonable, but not yet universally outcome‐proven adjunct until such data are available.

## 5 Conclusion

IVUS guidance in patients with ULMCA disease undergoing PCI demonstrated numerically favorable but statistically nonsignificant effects on mortality and repeat revascularization, without apparent differences in myocardial infarction or stent thrombosis. The large modern OPTIMAL trial significantly redefined the evidence base and dampened previous indications to support the use of IVUS routinely. In general, the existing randomized data suggest that IVUS is a procedurally useful adjunct, but fails to demonstrate consistent clinical superiority in all ULMCA presentations. Future adequately powered studies must not be limited to routine‐versus‐no‐routine comparisons but must outline a more individualized IVUS approach by determining the patient, lesion, and procedural phenotype that are most likely to yield significant clinical benefit.

## Author Contributions

Abdelrahman Elhakim, Mostafa Salem, Hamza A. Abdul‐Hafez, and Osama Bisht conceived and designed the study. Mohammed A. Elbahloul, Abdelrahman M. Elettreby, Mohamed A. Alsaied, and Maab M. Saleh performed the literature search, study screening, and full‐text eligibility assessment. Mohammed A. Elbahloul, Abdelrahman M. Elettreby, Mohamed A. Alsaied, Hamza A. Abdul‐Hafez, and Maab M. Saleh extracted the data. Ghaith Hussein Ahmad Al Bataineh and Amer Al Manla contributed to risk‐of‐bias assessment, GRADE evaluation, and evidence interpretation. Abdelrahman M. Elettreby and Mohammed A. Elbahloul performed the statistical analysis and prepared the figures and tables. Abdelrahman Elhakim, Mostafa Salem, Amer Al Manla, and Osama Bisht provided methodological supervision and clinical interpretation. All authors contributed to drafting and/or critically revising the manuscript for important intellectual content. All authors reviewed and approved the final manuscript and agreed to be accountable for all aspects of the work.

## Funding

The authors have nothing to report.

## Ethics Statement

This is a secondary analysis of already published studies, and no ethical approval is required. The review was conducted in accordance with recognized principles, including the PRISMA (Preferred Reporting Items for Systematic Reviews and Meta‐Analyses) framework, to ensure the study's transparency, integrity, and ethical conduct.

## Consent

The authors have nothing to report.

## Conflicts of Interest

The authors declare no conflicts of interest.

## Supporting information

Supporting File

## Data Availability

Supporting data of this study are available from the corresponding author upon a reasonable request.
