# Supplementary material for: IVUS‐Guided Versus Angiography‐Guided PCI for Unprotected Left Main Coronary Artery Disease: A Systematic Review, Meta‐Analysis, and GRADE Assessment of Randomized Trials
Source: Clin Cardiol. 2026 Jun 10;49(6):e70367. doi: 10.1002/clc.70367 (PMC13250836; doi:10.1002/clc.70367)
Supplement: Supplementary file 1 — Supporting File [file CLC-49-e70367-s001.docx]

**Table of Contents**

| Supplementary Material | Description | Page |
| --- | --- | --- |
| Table S1 | Databases search strategies | 2 |
| Figure S1 | Funnel plot showing trim and fill test for all cause death outcome | 4 |
| Figure S2 | Funnel plot showing trim and fill test for MI outcome | 5 |
| Figure S3 | Funnel plot showing trim and fill test for Stent thrombosis outcome | 6 |
| Figure S4 | Forest plot of cardiac death | 7 |
| Figure S5 | Leave-one-out sensitivity analysis of cardiac death | 8 |
| Figure S6 | Forest plot of target lesion revascularization (TLR) | 9 |
| Figure S7 | Leave-one-out sensitivity analysis of target lesion revascularization (TLR) | 10 |
| Figure S8 | Forest plot of target vessel revascularization (TVR) | 11 |
| Figure S9 | Leave-one-out sensitivity analysis of target vessel revascularization (TVR) | 12 |
| Figure S10 | Doi plot assessing publication bias for cardiac death | 13 |
| Figure S11 | Doi plot assessing publication bias for target lesion revascularization (TLR) | 14 |
| Figure S12 | Doi plot assessing publication bias for target vessel revascularization (TVR) | 15 |
| Figure S13 | Funnel plot showing trim and fill test for cardiac death outcome | 16 |
| Figure S14 | Funnel plot showing trim and fill test for TLR outcome | 17 |
| Figure S15 | Funnel plot showing trim and fill test for TVR outcome | 18 |

**Table S1.** Search strategy for each database

| **Database** | **Search strategy** | **Keywords** | **Total results** |
| --- | --- | --- | --- |
| PubMed | ("IVUS"[All Fields] OR ("intravascular"[All Fields] AND ("diagnostic imaging"[MeSH Subheading] OR ("diagnostic"[All Fields] AND "imaging"[All Fields]) OR "diagnostic imaging"[All Fields] OR "ultrasound"[All Fields] OR "ultrasonography"[MeSH Terms] OR "ultrasonography"[All Fields] OR "ultrasonics"[MeSH Terms] OR "ultrasonics"[All Fields] OR "ultrasounds"[All Fields] OR "ultrasound s"[All Fields])) OR ("intravascular"[All Fields] AND ("image"[All Fields] OR "image s"[All Fields] OR "imaged"[All Fields] OR "imager"[All Fields] OR "imager s"[All Fields] OR "imagers"[All Fields] OR "images"[All Fields] OR "imaging"[All Fields] OR "imaging s"[All Fields] OR "imagings"[All Fields])) OR ("ultrasonography, interventional"[MeSH Terms] OR ("ultrasonography"[All Fields] AND "interventional"[All Fields]) OR "interventional ultrasonography"[All Fields] OR ("intravascular"[All Fields] AND "ultrasonography"[All Fields]) OR "intravascular ultrasonography"[All Fields]) OR "IVUS-Guided"[All Fields]) AND ("PCI"[All Fields] OR ("percutaneous coronary intervention"[MeSH Terms] OR ("percutaneous"[All Fields] AND "coronary"[All Fields] AND "intervention"[All Fields]) OR "percutaneous coronary intervention"[All Fields]) OR ("stent s"[All Fields] OR "stentings"[All Fields] OR "stents"[MeSH Terms] OR "stents"[All Fields] OR "stent"[All Fields] OR "stented"[All Fields] OR "stenting"[All Fields])) AND (("left"[All Fields] AND "main"[All Fields] AND ("coronary vessels"[MeSH Terms] OR ("coronary"[All Fields] AND "vessels"[All Fields]) OR "coronary vessels"[All Fields] OR ("coronary"[All Fields] AND "artery"[All Fields]) OR "coronary artery"[All Fields])) OR "LMCA"[All Fields]) | All Fields | 708 |
| Cochrane | (IVUS OR intravascular ultrasound OR intravascular imaging OR intravascular ultrasonography OR IVUS-Guided) AND (PCI OR Percutaneous Coronary Intervention OR stent) AND (left main coronary artery OR LMCA) | Title Abstract Keyword | 105 |
| Web of Science | (IVUS OR intravascular ultrasound OR intravascular imaging OR intravascular ultrasonography OR IVUS-Guided) AND (PCI OR Percutaneous Coronary Intervention OR stent) AND (left main coronary artery OR LMCA) | Topic | 710 |
| Scopus | (IVUS OR “intravascular ultrasound” OR “intravascular imaging” OR “intravascular ultrasonography” OR IVUS-Guided) AND (PCI OR “Percutaneous Coronary Intervention” OR stent) AND (“left main coronary artery” OR LMCA) | Title, abstract, Keywords | 596 |

* No additional Filters were applied


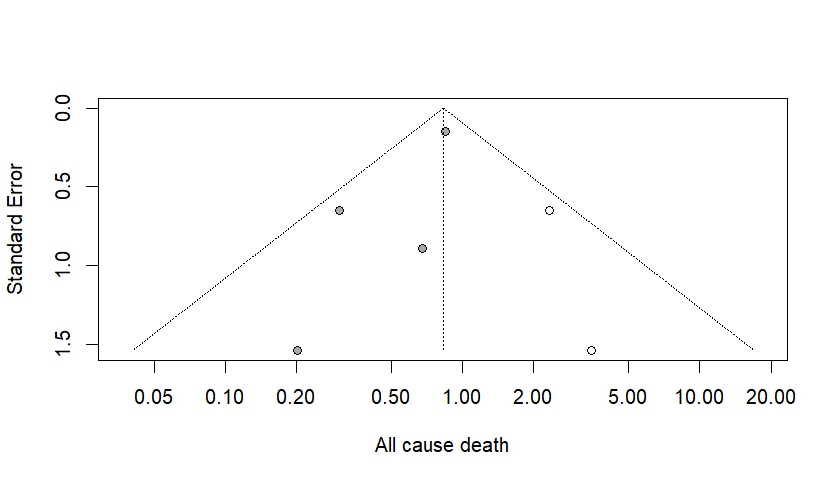


**Figure S1.** Funnel plot showing trim and fill test for all cause death outcome


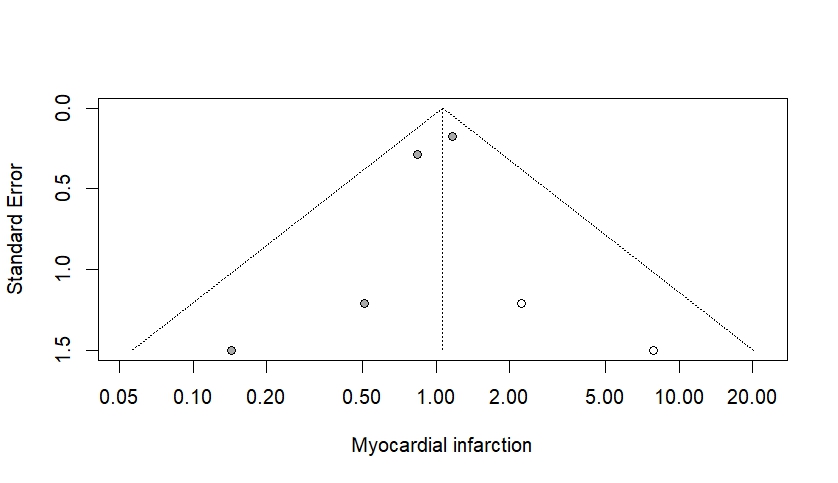


**Figure S2.** Funnel plot showing trim and fill test for MI outcome


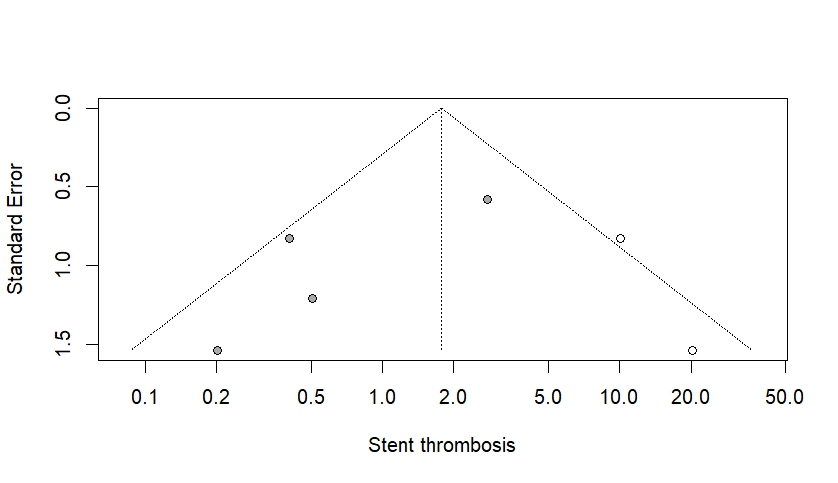


**Figure S3.** Funnel plot showing trim and fill test for Stent thrombosis outcome


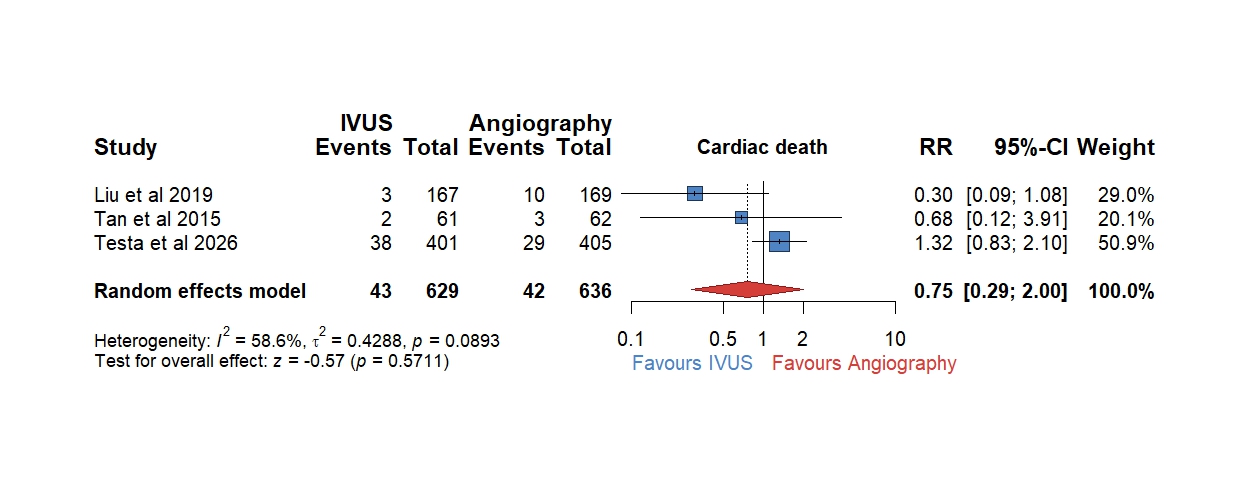


**Figure S4.** Forest plot of cardiac death comparing intravascular ultrasound-guided versus angiography-guided left main coronary artery intervention.


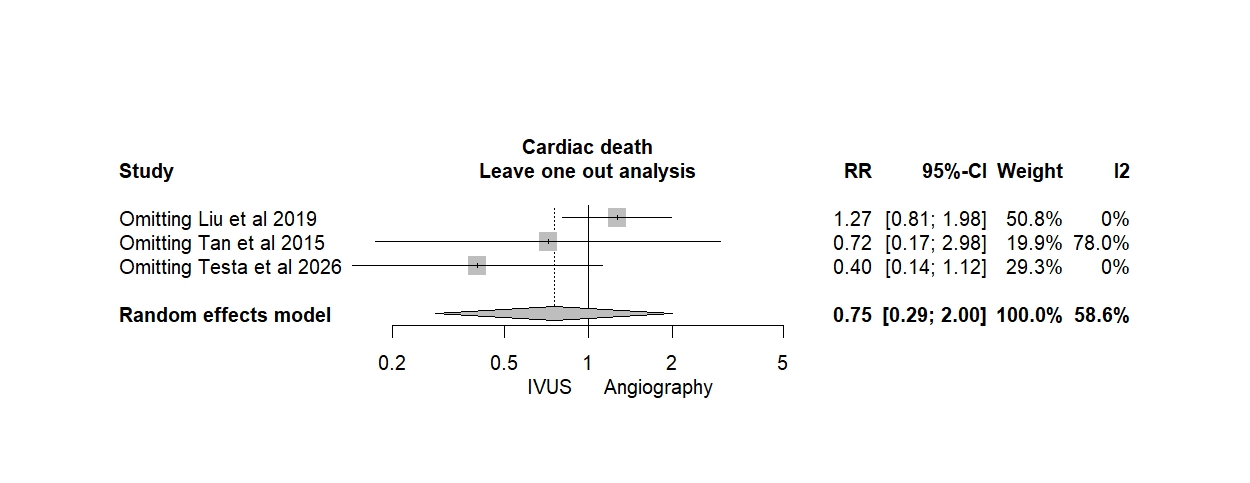


**Figure S5.** Leave-one-out sensitivity analysis of cardiac death.


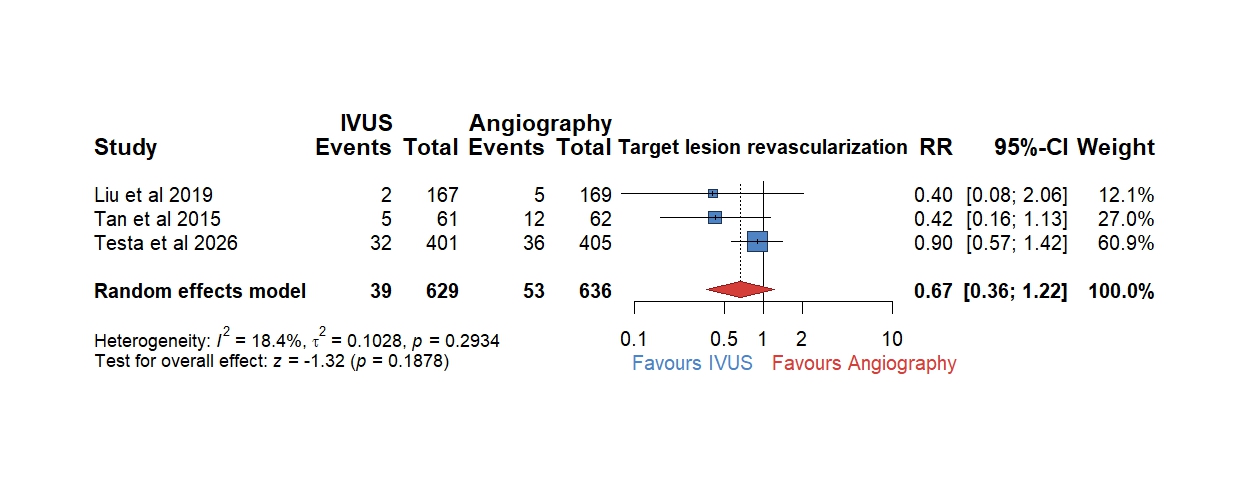


**Figure S6.** Forest plot of target lesion revascularization comparing intravascular ultrasound-guided versus angiography-guided left main coronary artery intervention.


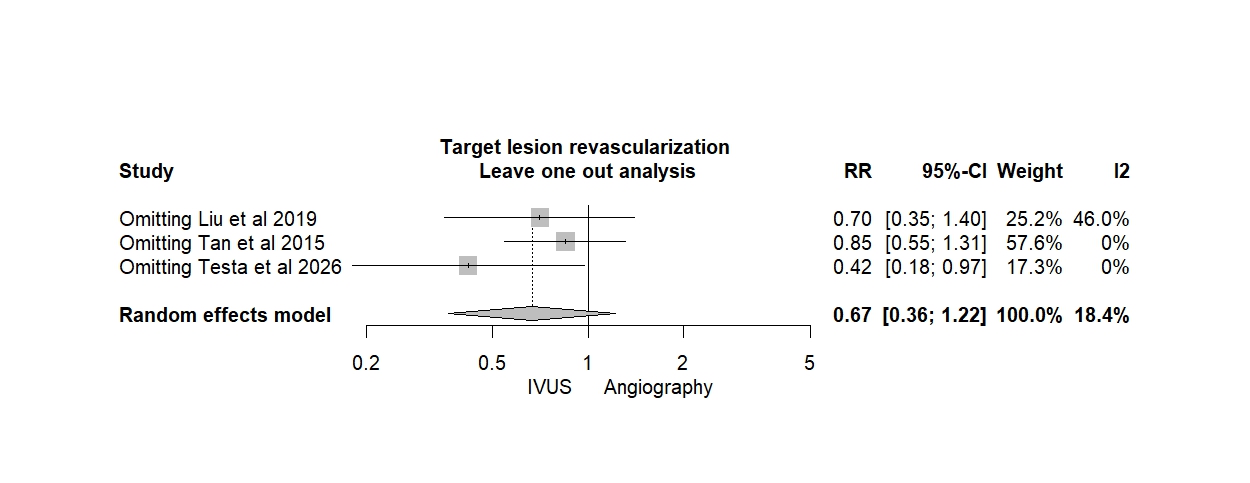


**Figure S7.** Leave-one-out sensitivity analysis of target lesion revascularization.


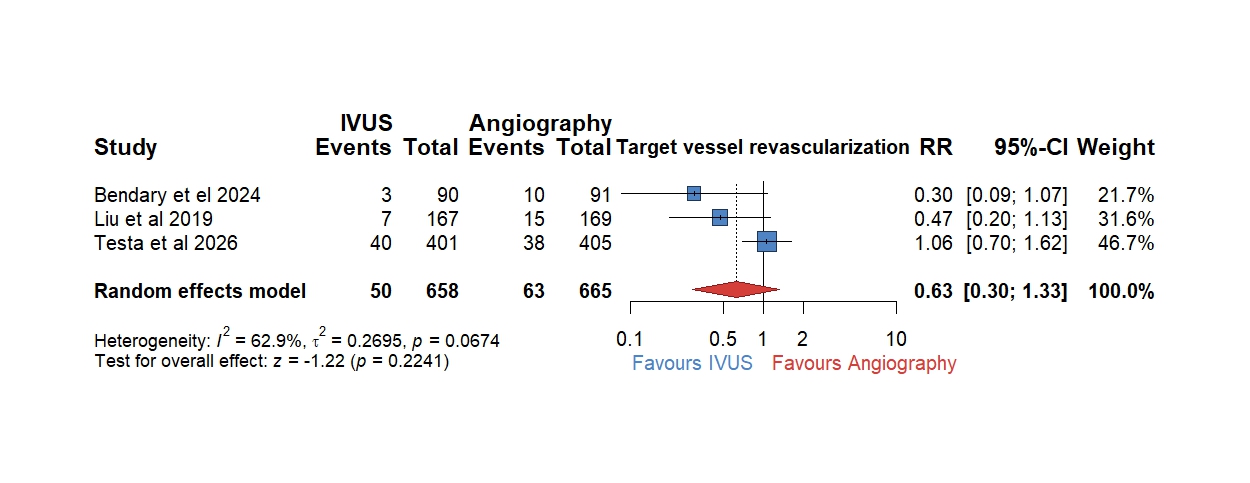


**Figure S8.** Forest plot of target vessel revascularization comparing intravascular ultrasound-guided versus angiography-guided left main coronary artery intervention.


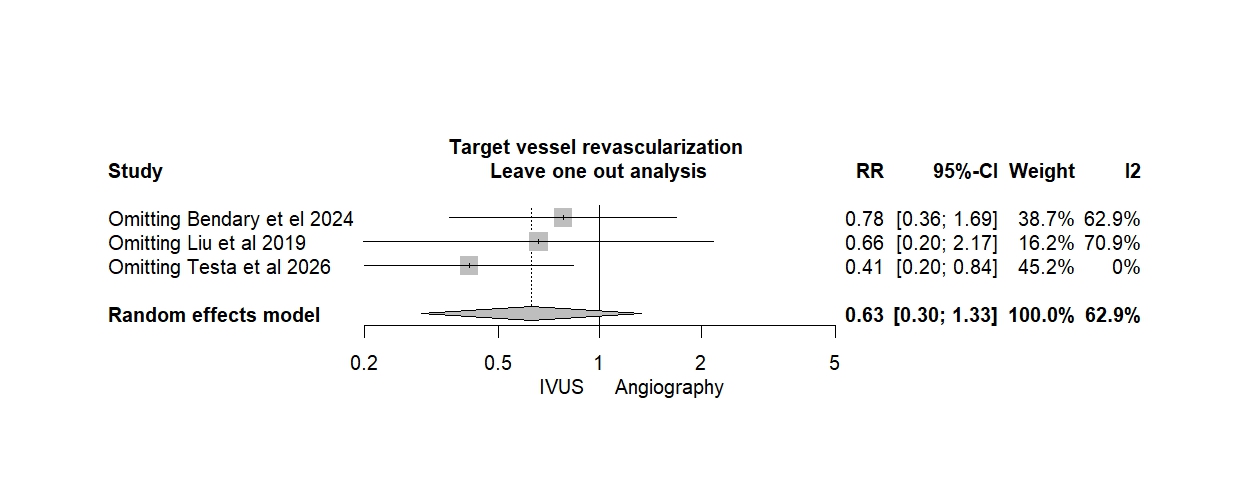


**Figure S9.** Leave-one-out sensitivity analysis of target vessel revascularization.


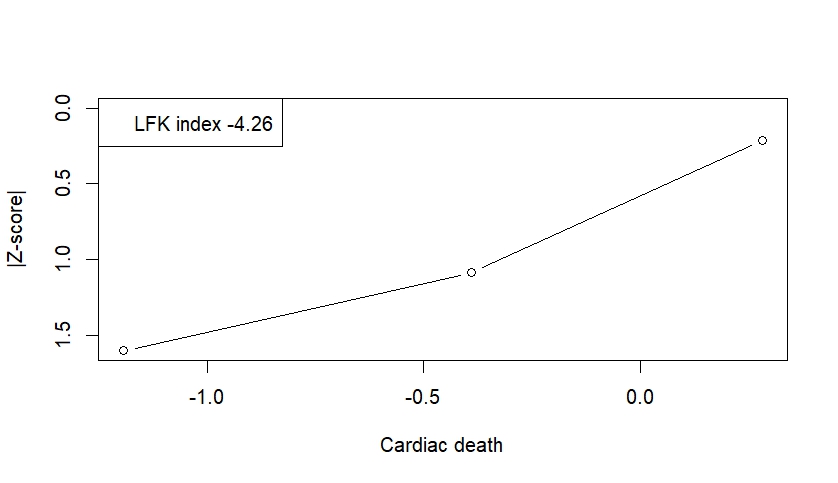


**Figure S10.** Doi plot assessing publication bias for cardiac death.


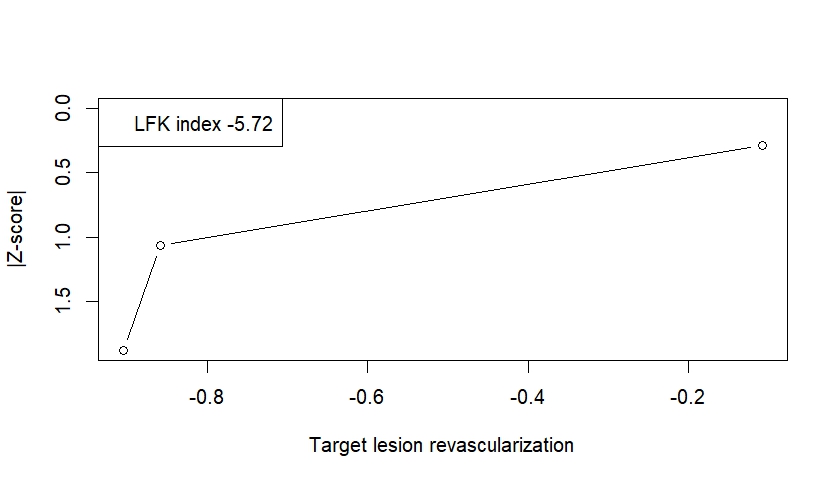


**Figure S11.** Doi plot assessing publication bias for target lesion revascularization.


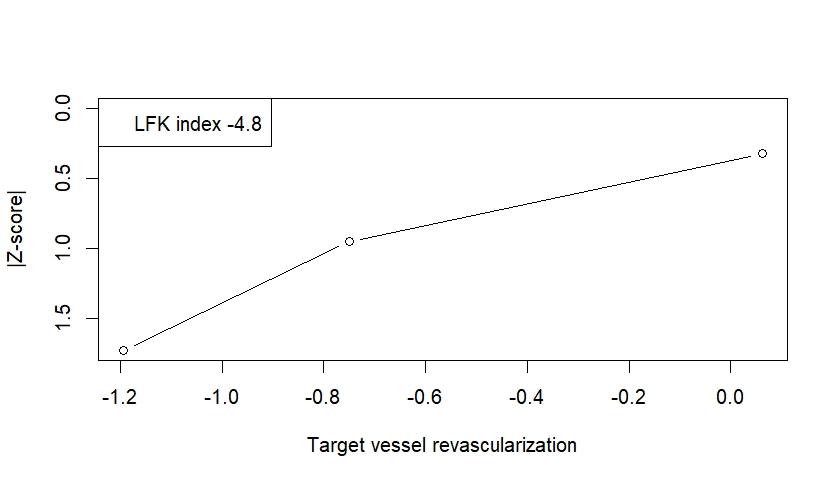


**Figure S12.** Doi plot assessing publication bias for target vessel revascularization.


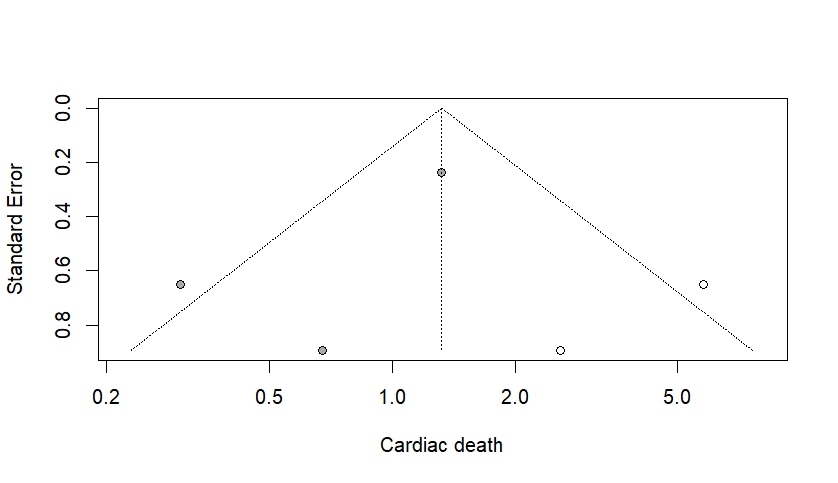


**Figure S13.** Funnel plot showing trim and fill test for cardiac death outcome


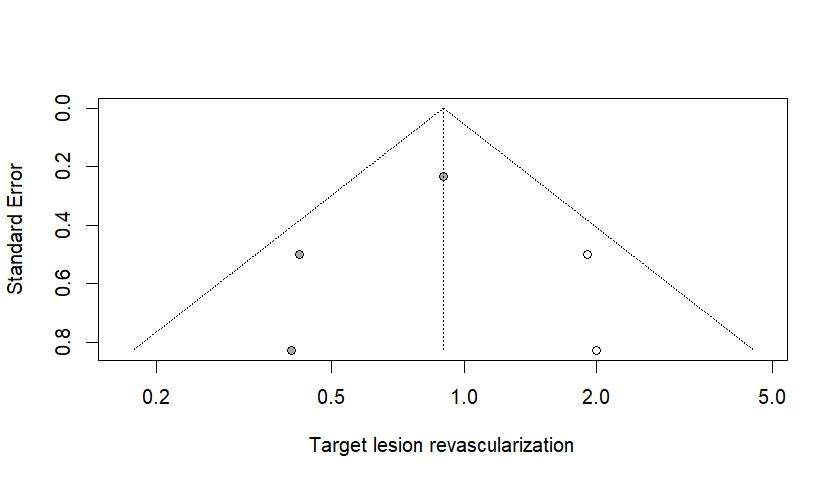


**Figure S14.** Funnel plot showing trim and fill test for TLR outcome


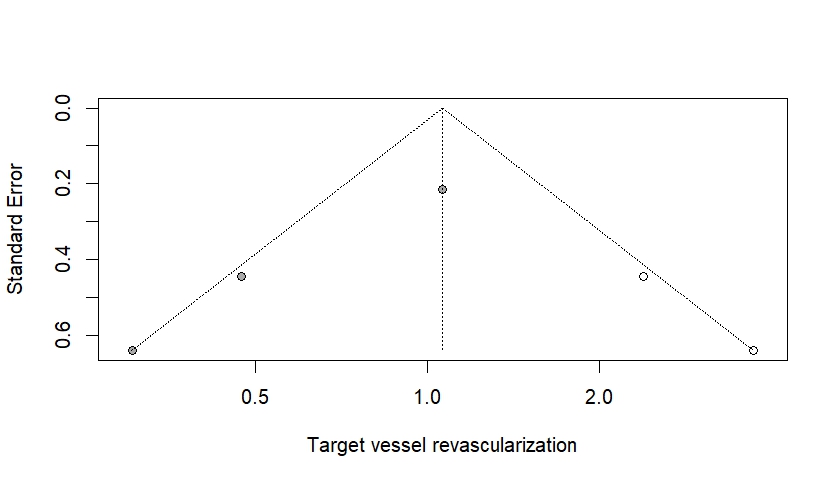


**Figure S15.** Funnel plot showing trim and fill test for TVR outcome
